# Physcraper: a Python package for continually updated phylogenetic trees using the Open Tree of Life

**DOI:** 10.1186/s12859-021-04274-6

**Published:** 2021-06-29

**Authors:** Luna L. Sánchez-Reyes, Martha Kandziora, Emily Jane McTavish

**Affiliations:** 1 School of Natural Sciences, University of California, Merced, USA; 2 Department of Botany, Faculty of Science, Charles University, Prague, Czech Republic

**Keywords:** Gene tree, Gene phylogeny, Multilocus, Interoperability, Open science, Reproducibility, Public database, DNA alignment, Open Tree of Life, Otol

## Abstract

**Background:**

Phylogenies are a key part of research in many areas of biology. Tools that automate some parts of the process of phylogenetic reconstruction, mainly molecular character matrix assembly, have been developed for the advantage of both specialists in the field of phylogenetics and non-specialists. However, interpretation of results, comparison with previously available phylogenetic hypotheses, and selection of one phylogeny for downstream analyses and discussion still impose difficulties to one that is not a specialist either on phylogenetic methods or on a particular group of study.

**Results:**

Physcraper is a command-line Python program that automates the update of published phylogenies by adding public DNA sequences to underlying alignments of previously published phylogenies. It also provides a framework for straightforward comparison of published phylogenies with their updated versions, by leveraging upon tools from the Open Tree of Life project to link taxonomic information across databases. The program can be used by the nonspecialist, as a tool to generate phylogenetic hypotheses based on publicly available expert phylogenetic knowledge. Phylogeneticists and taxonomic group specialists will find it useful as a tool to facilitate molecular dataset gathering and comparison of alternative phylogenetic hypotheses (topologies).

**Conclusion:**

The Physcraper workflow showcases the benefits of doing open science for phylogenetics, encouraging researchers to strive for better scientific sharing practices. Physcraper can be used with any OS and is released under an open-source license. Detailed instructions for installation and usage are available at https://physcraper.readthedocs.io.

## Background

Phylogenies capture the shared history of organisms and provide key evolutionary context for our biological observations [1]. Updating existing phylogenies with publicly available molecular sequence data provides the opportunity to simultaneously study the evolutionary history of many taxa in a reproducible and continuous manner. Increased taxonomic sampling is known to improve phylogenetic reconstructions [2, 3], time of divergence estimates [4, 5], and biogeographic analyses [6], as well as help in resolving phylogenetic conflict [3, 7, 8]. Here, we introduce Physcraper, a Python package that provides a data interoperability framework to automate data connections across biological databases, with the main goal of building upon published alignments and phylogenies to extend existing phylogenetic inferences with sequence data available for more taxa in public DNA databases. Physcraper links tip taxon labels in the updated trees to a unified taxonomic resource [9], effectively streamlining connections between phylogenies and any type of biological data.

Information such as geographical location, fossil ranges, and genetic and phenotypic data increasingly available in public biological databases constitute an amazing resource for scientific discovery [10]. One of the main challenges for automatic integration of data across biological databases are varying taxonomic idiosyncrasies. To address this challenge, the Open Tree of Life project (OpenTree) created a unified taxonomy for automatic taxonomic name standardization, by integrating taxonomic data from several resources [9], including the USA National Center for Biodiversity Information (NCBI) taxonomy [11, 12], and the Global Biodiversity Information Facility (GBIF) [13], among many others. OpenTree’s unified taxonomy, along with tools and methods to manage it, are available as Application Programming Interfaces (APIs), which are implemented as open access services for the general public [14]. Physcraper leverages on existing OpenTree’s unified taxonomy APIs to automatically standardize taxon names in any phylogeny, a key step to streamline the connection of updated phylogenies with data from different and independent biological databases.

Decades of single locus sequencing have generated massive amounts of homologous DNA datasets that have the potential to be used for phylogenetic reconstruction at many scales [15]. More than a decade ago, GenBank release 159 (April 15, 2007) already hosted 72 million DNA sequences that were gauged to have the potential to resolve phylogenetic relationships of 98.05% of the almost 241,000 distinct taxa in the NCBI taxonomy at the time [15]. However, even thirteen years later, phylogenetic estimates for most of these taxa are still not available [16]. OpenTree assembles a comprehensive synthetic tree of life comprising 2.3 million tips, of which around 90,000 are supported by publicly available expert phylogenetic data that has been uploaded to OpenTrees’ database (the Phylesystem [17]) by volunteer curators—the remaining 1.4 million taxa are placed in the synthetic tree based on OpenTree’s unified taxonomy. There is a considerable amount of phylogenetically informative data in GenBank with the potential to fill these phylogenetic gaps in the tree of life, but this data either has not been analysed or the analyses have not been made publicly available and accessible [16].

Assembling a DNA alignment from a massive database such as GenBank can be done “by hand”, but that is a time-consuming approach which is largely non reproducible. Various computational pipelines that mine DNA databases fast, efficiently, and reproducibly have been developed and widely used to infer phylogenetic relationships of many organisms (e.g., [18–21]). While genomics has, and will continue to revolutionize phylogenetic inference, the diversity of alternative genomic sequencing approaches that are implemented produce widely non-overlapping homology hypotheses across taxa, creating challenges for phylogenetic reconstruction [22]. Phylogenomics addresses this problem by focusing on targeted capture of informative regions [23]. However, carefully curated markers and alignments can significantly improve phylogenetic reconstructions, even in phylogenomic analyses [24].

Physcraper improves on previous work that automates phylogenetic reconstruction, by leveraging on the knowledge contained in existing homology hypotheses that phylogeneticists and taxon specialists have assessed and deemed appropriate for a specific phylogenetic scope. There are almost 8,200 publicly available, peer-reviewed curated alignments, covering around 100,000 distinct taxa in the TreeBASE database [25–27], which can be used as seeds to mine molecular databases, and as “jump-start” alignments for phylogenetic reconstruction [28] to continually enrich, update and compare phylogenetic hypotheses to existing evolutionary knowledge.

Physcraper is implemented as a Python pipeline that uses OpenTree’s APIs to automatically link any phylogeny mapped to OpenTree’s unified taxonomy, to alignments from TreeBASE, and data from GenBank. Physcraper’s usage and functionalities are presented with a case-study analysis of a group of flowering plants, the hollies.

## Implementation

Physcraper is implemented with Python and can be run on a Python interactive session, as a Python script, or using the command line interface we developed for it. It currently consists of 13 modules. For testing and improving Physcraper’s Python code syntax quality, we used the Pylint software following instructions from its website [29] and manual [30], with a “.pylintrc” configuration file. As of now, all Physcraper modules have a Pylint score of 10/10.

**Fig. 1:**
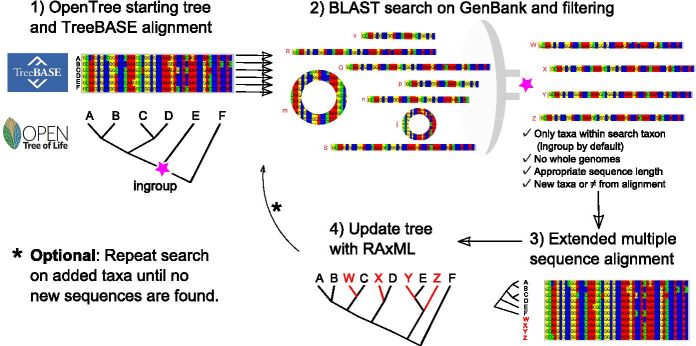
The Physcraper framework consists of four general steps. The star represents the “search taxon”, the Most Recent Common Ancestor (MRCA) of all taxa in the ingroup that is also a named clade in the NCBI taxonomy. The software is fully described on its documentation website at https://physcraper.readthedocs.io, along with installation instructions, function usage descriptions, examples and tutorials

The general Physcraper framework (Fig. 1) consists of 4 steps: (1) identifying and processing a phylogeny and its underlying alignment; (2) performing a BLAST search of DNA sequences from original alignment on GenBank, and filtering of new sequences; (3) profile-aligning new sequences to original alignment; (4) performing a phylogenetic analysis and comparing the updated results to existing phylogenies.

### The inputs: a phylogeny and an alignment

Taxon names in the input phylogeny must be standardized to OpenTree’s unified taxonomy [31] using OpenTree’s bulk Taxonomic Name Resolution Service (TNRS) tool [32]. Users can upload their own phylogeny, or choose from among the 2, 950 curated phylogenies stored in OpenTree’s Phylesystem database [33] that also have alignments available on the TreeBASE database [34, 35].

The input alignment is a single locus DNA dataset that was used in part or in whole to generate the input phylogeny. Physcraper retrieves TreeBASE alignments automatically. Alternatively, users can provide the path to a local copy of the alignment of their choosing. Only taxa that are both in the sequence alignment and in the phylogeny are considered further for analysis; at least one taxon and its corresponding sequence are required.

### DNA sequence search and filtering

The Basic Local Alignment Search Tool, BLAST [36] is used for DNA sequence search either on a remote or a local GenBank database. It is constrained to a “search taxon”, which corresponds to the Most Recent Common Ancestor (MRCA) of all ingroup taxa that is also a named clade in the NCBI taxonomy (Fig. 1). The search taxon is identified using OpenTree’s unified taxonomic API [37].

BLAST is performed using the blastn algorithm [38] implemented in BioPython 1.71 [39] NCBIWWW module [40] modified to accept an alternative BLAST address. Each sequence in the alignment is BLASTed once against the GenBank database. Matching sequences are filtered and excluded from the analysis if they (1) are not in the search taxon; (2) have an e-value above the cutoff (default to 0.00001); (3) fall outside a minimum and maximum sequence length threshold, defined as a proportion of the average sequence length without gaps of all sequences in the input alignment (default values of 80% and 120%, respectively); (4) or, if they are either identical to or shorter than an already existing sequence in the input alignment, and they represent the same taxon in NCBI’s or OpenTree’s unified taxonomy. By default, an arbitrary maximum number of 5 sequences per taxon are chosen at random from the set of matching sequences that passed the filtering step.

Reverse, complement, and reverse-complement sequences are identified and translated using BioPython internal functions [39]. Iterative cycles of BLAST searches can be performed, by blasting all new sequences until no new ones are found. By default only one BLAST cycle is performed.

### New DNA sequence alignment

MUSCLE [41] is used to perform a profile alignment in which the original alignment is used as a template of homology criteria to align new sequences. The final alignment is not further automatically checked, and additional inspection and refinement are recommended.

### Phylogenetic reconstruction and comparison

RAxML [42] is implemented to reconstruct a Maximum Likelihood (ML) gene phylogeny for each input alignment with default settings (GTRCAT model and 100 bootstrap replicates with default algorithm), using the input phylogeny as starting tree for ML searches. Bootstrap results are summarized using DendroPy’s SumTrees module [43].

Physcraper’s main result is an updated phylogenetic hypothesis for the search taxon. Updated and original phylogenies are compared with Robinson-Foulds weighted and unweighted metrics calculated with Dendropy [43], and with a node by node comparison between the synthetic OpenTree and the original and updated phylogenies individually, using OpenTree’s conflict API [44].

## Results

### Case study: the hollies

A user is interested in phylogenetic relationships within the genus *Ilex*. Commonly known as “hollies”, the genus encompasses between 400 [45] and 500 recognized living species [46], and is the only extant taxon within the family Aquifoliaceae, in the order Aquifoliales of flowering plants [47].

An online literature review in June 2020 (Google scholar search for “ilex phylogeny”) revealed that there are several published studies addressing phylogenetic relationships within the hollies [45, 48–52], but only the “Gottlieb2005” study [45] and the “Yao2020” study [52] have data openly available. The Gottlieb2005 phylogeny and alignment are available in TreeBASE study 1091 [53]. The Gottlieb2005 phylogeny samples 41 species, is available in OpenTree’s Phylesystem (study pg_2827 [54]), and has been integrated into OpenTree’s synthetic tree [55]. The Yao2020 *Ilex* phylogeny is the most recent one for the genus [52], and it is only available in OpenTree’s Phylesystem (study ot_1984 [56]), and in the DRYAD repository [57]. With 175 tips, the Yao2020 phylogeny [52] is the best sampled phylogeny available for the genus *Ilex*. In order to showcase Physcraper’s performance, we chose the Gottlieb2005 phylogeny and a corresponding single locus alignment of the internal transcribed spacer DNA region (ITS) as Physcraper inputs, to update relationships in the genus *Ilex*. Currently being the best sampled and most recent phylogenetic hypothesis for *Ilex*, we used the Yao2020 phylogeny as the ideal standard to compare results from this Physcraper case study.

**Fig. 2:**
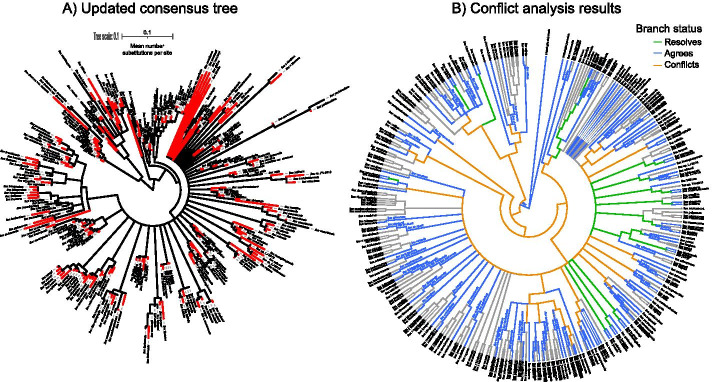
**a** Phylogeny updated with Physcraper using a starting phylogeny and an alignment from [45] (Gottlieb2005 data in text). Tips in original alignment and new tips added with Physcraper are depicted in black and red, respectively. First, Physcraper obtained sequences from the GenBank database via local BLAST of all sequences in the Gottlieb2005 original alignment of the internal transcribed spacer DNA region (ITS). Then, it filtered the obtained sequences following criteria described in section “DNA sequence search and filtering”, and aligned them to the original Gottlieb2005 alignment using MUSCLE. Finally, Physcraper performed a phylogenetic reconstruction using RAxML, with 100 bootstraps. **b** Results of conflict analysis performed using OpenTree’s conflict tool [44]. The Physcraper updated Gottlieb2005 phylogeny in **a** was compared to an *Ilex* OpenTree synthetic subtree v. 12.3 [72] constructed using taxonomy of the genus as backbone and resolving branches based on phylogenetic data from the original Gottlieb2005 phylogeny. Green branches are resolved by the updated phylogeny, blue branches are in agreement between the updated phylogeny and the synthetic subtree, and orange branches are in conflict between the two

We ran Physcraper on a computer node of the Multi-Environment Research Computer for Exploration and Discovery (MERCED) Linux cluster from the University of California, Merced, using one core at 2301 MHz RAM. BLAST and RAxML analyses ran for 19hrs 45min, with bootstrap analyses taking an additional 13hrs. The Gottlieb2005 phylogeny [45] updated using Physcraper (Fig. 2; Physcraper updated phylogeny from now on) displays all 41 distinct taxa from the original study plus 231 new tips, contributing phylogenetic data to 84 additional *Ilex* taxa. The best ML phylogeny from the RAxML analysis is 99% resolved, with 25% of nodes with bootstrap support < 0.1 and 48% nodes with bootstrap support > 0.75 (Fig. 2a). A large portion of internal branches are negligibly small, with 30 branches < 0.00001 substitution rate units, from which only 9 have a bootstrap support > 0.75 (Fig. 2). As comparison with the Physcraper updated phylogeny, the Yao2020 phylogeny [52] also contains all 41 distinct taxa sampled in the Gottlieb2005 phylogeny [45], while contributing phylogenetic data to 134 additional *Ilex* taxa. From these, 67 taxa are also in the Physcraper updated phylogeny. While the Yao2020 phylogeny [52] was also constructed using ITS as a marker, their GenBank data is not released yet. Hence, Physcraper was unable to incorporate 68 taxa that are only on the Yao2020 phylogeny because the DNA data is unavailable. We also note that Physcraper incorporates 18 *Ilex* taxa that are not in the Yao2020 phylogeny [52]. These taxa appear nested among other *Ilex* species (Fig. 2b) and visual inspection of the DNA sequences suggests they are correctly assigned as *Ilex*. The ITS alignment that underlies the Yao2020 phylogeny was constructed without any tool to mine GenBank [52], which could explain why Physcraper was able to incorporate these 18 additional *Ilex* taxa in the Physcraper updated phylogeny.

### Verification test

To test the performance of Physcraper we performed a verification test in which we pruned 9 out of the 41 tips in the original Gottlieb2005 phylogeny [45], corresponding to a 20% trim, excluding the outgroups. We then performed a Physcraper run to test if we would recover the pruned tips. To perform the BLAST searches, Physcraper automatically drops sequences from the alignment belonging to taxa absent from the input phylogeny. The Physcraper updated phylogeny successfully recovered only 6 out of 9 pruned tips. Closer examination of results revealed that sequences for the 3 missing tips were correctly retrieved with BLAST along with the 6 sequences belonging to the remaining pruned tips, but were excluded from the alignment step. We then followed the GenBank accession numbers reported in [45] belonging to the sequences of the 3 missing tips, *Ilex warburgii* (original accession number reported: U92600/U92601; updated: AH007153.2), *Ilex dimorphophylla* (original accession number reported: U92592/U92593; updated: AH007149.2), and *Ilex percoriacea* (accession number: AH007156.2). We note that these three sequences contain a 100 bp long gap of unidentified nucleotides (Ns) that is completely absent from the original alignment. This caused them to exceed Physcraper’s default sequence length cutoff of 120%, being thus filtered and excluded from further analyses. The missing ITS sequences do appear in Physcraper’s output file “seqlen_mismatch.txt”, that includes the accession number, taxon name, and sequence length of all BLAST matches that were filtered based on sequence length cutoffs set in the configuration file.

## Discussion

Databases preserving and democratizing access to biological data have become essential resources for science. New molecular data keep accumulating and tools facilitating its integration into existent evolutionary knowledge contribute to the acceleration of scientific discovery. Physcraper is a tool that builds upon previous knowledge stored in published alignments and phylogenies, taking advantage of OpenTree’s services to facilitate comparison of phylogenies, with the main goal of extending our knowledge of phylogenetic relationships across the tree of life. We believe this is a key step to successfully establish an open, reproducible workflow for phylogenetics. As such, it facilitates access to phylogenetic knowledge for non-specialists in diverse research areas (e.g., ecology, medicine), effectively contributing to the democratization of phylogenetic studies.

As a tool for automatizing phylogenetic reconstruction from molecular databases, Physcraper presents several advantages over existing phylogenetic pipelines designed to make evolutionary sense of the vast amount of public genetic data available. Unlike phylogenetic placement approaches [58, 59], which add new taxa without modifying the input phylogeny, Physcraper estimates all the relationships anew in the context of the new data. The tool PUMPER [20] shares these conceptual strengths, but is no longer under active development, is challenging to install and run, and has produced few published phylogenetic analyses since its publication. Several other existing tools create full phylogenies *de novo* by mining of molecular databases [15, 19, 21, 60, 61]. In particular, Phylota [15], and PHLAWD [18], have been cited and used abundantly. Physcraper adds to this automated database mining concept by incorporating prior phylogenetic work and existing taxonomic domain knowledge on appropriate markers and alignment construction, to update existing phylogenetic knowledge. This decreases error (requiring less manual downstream processing) and eases comparison with previous phylogenetic hypotheses. Results from the verification test highlight the importance of incorporating existing expertly curated homology statements to automatically update phylogenetic relationships, instead of ignoring the information they contain and building homology statements fully *de novo*.

We encourage users to look at Physcraper’s output files containing information about the filtered sequences, and use this results to potentially modify configuration parameters such as the sequence length cutoff parameter in subsequent Physcraper runs. Physcraper’s default filtering parameters are arbitrary, but we hope that by making the process of locating homologous sequences online reproducible, and tracking what filters are used, we make it easier for researchers to delve into the effect that different filtering choices have on their inferences. This is in contrast to “manual” searches for DNA sequences in molecular databases, where similar arbitrary filters are applied, but are difficult to trace. It has been shown [62] that the effect of missing data in alignments can be enigmatic, and interact with the true phylogenetic relationships for the dataset at hand. There is not currently strong support in the literature for any particular filtering cutoff value, and rather than prescribe specific approaches, we encourage users to explore the effects of different parameter values on resulting phylogenetic hypotheses. In addition, by providing the output files at each step of the analysis, it is straightforward to assess how changing filtering cutoffs and software choices might drive differences in phylogenetic inference. By gathering the DNA sequences, and making the unaligned files easily accessible and reusable, Physcraper also facilitates the exploration of alternative aligning tools. Once sequences are aligned, users can apply practically any phylogenetic software and compare results using Physcraper tools in a reproducible framework.

Organellar genome sequences, such as chloroplasts and mitochondria will also generally be excluded from automatic addition based on default Physcraper length cutoffs. Multiple sequence alignment of loci of drastically different lengths is unfeasible, and we have found in testing that it often returns incorrect results, splitting shorter sequences with many long gaps to align with exact matches across the entire longer locus. While it would be possible to directly extract the BLAST match from genomes, this would exclude potentially homologous flanking regions which are not matched by BLAST’s local search algorithm, but that may be important for phylogenetic inference. Instead we list the accession numbers for these matches in the “seqlen_mismatch.txt” file for users to assess and incorporate appropriate homologous regions to their alignment of interest.

Physcraper generates gene trees, which individually do not capture the full complexity of species’ evolutionary history [63]. In addition, single gene phylogenies with very high numbers of taxa may lack sufficient signal for accurate phylogenetic resolution [64]. The Physcraper workflow avoids this challenge by focusing on ingroup taxa of an existing phylogeny, using markers that have been assessed and proven appropriate for that phylogenetic scope in past publications. Also, Physcraper thins alignments by removing sequences identical to original and newly added sequences, and by setting a maximum number of sequences per taxon. Nonetheless, it is incumbent on users to assess their final inference with respect to statistical support and biological plausibility.

In the era of phylogenomics, rigorous analyses of multiple loci still allow for more complex evolutionary models than analyses of large genomic data sets, and in many cases can provide better evolutionary estimates. For example [65] show that when applying coalescent models, there is more information in two genes of 300 bp each than in 600 independent sites. Physcraper is designed to facilitate gathering alignments and gene trees for multiple loci from a group of interest, that together can be used to reconstruct species trees taking into account coalescent processes with ASTRAL [66] or SVD Quartets [67]. Physcraper’s “multi_locus.py” module allows to automatically merge the outputs of Physcraper runs from different loci into input files for the two software mentioned above, or as concatenated alignments for supermatrix analyses.

Our case study application of Physcraper to update a phylogeny of the genus *Ilex* is based on a single marker, so we expect for it to be not as well resolved as phylogenies resulting from analyses that used multiple markers. Although not perfect, the Physcraper updated *Ilex* phylogeny seems biologically reasonable in different ways. All samples corresponding to the ingroup are clustered together, forming a monophyletic group (Fig. 2a), and samples belonging to the same *Ilex* species also form monophyletic groups (Fig. 2b). A notable exception are samples of the species *Ilex theeizans*, which appear as non-monophyletic in the updated phylogeny as well as in the original Gottlieb2005 phylogeny. A visual comparison of the Yao2020 phylogeny and the original Gottlieb2005 phylogeny suggests that relationships within the genus *Ilex* are still being actively determined, and that increased taxon sampling might be key to resolve them.

Physcraper has the added advantage of facilitating the linkage of taxonomic information about tips in the output phylogenies to data available in a variety of biological databases [9], such as geographical locations for taxa from the GBIF [13]. Taxonomic links, and comparisons to existing published phylogenies in the OpenTree data store can also help flag paralogous sequences. Accidentally including paralogs as homologs is a known risk of assembling a dataset for phlyogenetic analysis, and can be more prevalent in automatically assembled datasets than in manually curated ones. We provide users with several tools to assess homology of aligned sequences. The estimated gene phylogeny itself is an evolutionarily explicit way to visualize gene evolution, which in concert with taxonomic labelling can reveal paralogy. OpenTree’s conflict analysis tool implemented in Physcraper informs the users of whether their phylogeny contains major conflicts with established taxonomy and any phylogenetic context they wish to compare to. This tool also returns information on taxonomic and phylogenetic conflicts that exist in the original input phylogeny. Detected conflicts may be a sign that taxonomy needs to be updated, or may be a sign that non-homologous sequences have been included in the analysis. These taxonomic and phylogenetic conflicts flag regions of the phylogeny for the researcher to more closely examine and assess homology.

The Physcraper workflow can be used to rapidly (in a matter of hours) create phylogenies which can address challenges overarching both fields of ecology and evolution, such as phylogenetically placing newly discovered species [68], curating taxonomic assignments [69], and generating custom trees for ecological [70] and evolutionary downstream analyses [71].

## Conclusions

Data repositories hold more information than meets the eye. Beyond the main data, they are rich sources of metadata that can be leveraged for the advantage of all areas of biology as well as the advancement of scientific policy, applications and education. Scientific understanding is constantly challenged and reframed by new data and analyses. Physcraper provides a framework for reproducible phylogenetics that has the potential to consistently contextualize new knowledge in the light of previous understanding, showcasing the utility and importance of good data sharing practices and open science for the advancement of phylogenetics, biology and research.

## Availability and requirements

- **Project name:** Physcraper
- **Project home page:** https://physcraper.readthedocs.io/en/latest/index.html
- **Operating System:** Linux, Mac, Windows
- **Programming Language:** Python
- **Other requirements:** Dependencies
- **License:** GNU
- **Any restrictions to use by non-academics:** As specified by the License

## Data Availability

Code and datasets developed and analysed for this study are available at the following GitHub repositories: “physcraper”—contains the source code, https://github.com/McTavishLab/physcraper; “physcraperex” – contains the examples, https://github.com/McTavishLab/physcraperex; and, “physcraper_ms”—contains this reproducible manuscript, https://github.com/McTavishLab/physcraper_ms.

## Abbreviations

OpenTree: The Open Tree of Life project
TNRS: Taxonomic Name Resolution Service
MRCA: Most Recent Common Ancestor
BLAST: Basic Local Alignment Search Tool
NCBI: USA National Center for Biodiversity Information
GBIF: Global Biodiversity Information Facility
API: Application Programming Interface

## References

[1] Dobzhansky T (1973). Nothing in biology makes sense except in the light of evolution. Am Biol Teach.

[2] Hillis DM (1996). Inferring complex phylogenies. Nature.

[3] Natsidis P, Tsakogiannis A, Pavlidis P, Tsigenopoulos CS, Manousaki T (2019). Phylogenomics investigation of sparids (Teleostei: Spariformes) using high-quality proteomes highlights the importance of taxon sampling. Commun Biol.

[4] Schulte JA. Undersampling taxa will underestimate molecular divergence dates: an example from the South American lizard clade Liolaemini. Int J Evol Biol. 2013. 10.1155/2013/628467 PMC3809987 24222886

[5] Soares AE, Schrago CG (2015). The influence of taxon sampling on Bayesian divergence time inference under scenarios of rate heterogeneity among lineages. J Theor Biol.

[6] Kayaalp P, Stevens MI, Schwarz MP (2017). Back to Africa: increased taxon sampling confirms a problematic Australia-to-Africa bee dispersal event in the Eocene. Syst Entomol.

[7] Hedtke SM, Townsend TM, Hillis DM (2006). Resolution of phylogenetic conflict in large data sets by increased taxon sampling. Syst Biol.

[8] Townsend JP, Lopez-Giraldez F (2010). Optimal selection of gene and ingroup taxon sampling for resolving phylogenetic relationships. Syst Biol.

[9] Rees JA, Cranston K (2017). Automated assembly of a reference taxonomy for phylogenetic data synthesis. Biodiversi Data J.

[10] Baxevanis AD, Bateman A (2015). The importance of biological databases in biological discovery. Curr Protoc Bioinform.

[11] Federhen S (2012). The NCBI taxonomy database. Nucl Acids Res.

[12] Schoch CL, Ciufo S, Domrachev M, Hotton CL, Kannan S, Khovanskaya R, Leipe D, Mcveigh R, O’Neill K, Robbertse B, Sharma S, Soussov V, Sullivan JP, Sun L, Turner S, Karsch-Mizrachi I. NCBI taxonomy: a comprehensive update on curation, resources and tools. Database. 2020. 10.1093/database/baaa062 PMC7408187 32761142

[13] GBIF Secretariat: GBIF Backbone Taxonomy. Checklist dataset. 10.15468/39omei. Accessed via GBIF.org on April 2021. https://www.gbif.org/dataset/d7dddbf4-2cf0-4f39-9b2a-bb099caae36c.

[14] OpenTreeOfLife, Redelings B, Cranston KA, Allman J, Holder MT, McTavish EJ. Open tree of life APIs V. 3.0. https://github.com/OpenTreeOfLife/germinator/wiki/Open-Tree-of-Life-Web-APIs.

[15] Sanderson MJ, Boss D, Chen D, Cranston KA, Wehe A (2008). The PhyLoTA browser: processing genbank for molecular phylogenetics research. Syst Biol.

[16] McTavish EJ, Drew BT, Redelings B, Cranston KA (2017). How and why to build a unified tree of life. BioEssays.

[17] McTavish EJ, Hinchliff CE, Allman JF, Brown JW, Cranston KA, Holder MT, Rees JA, Smith SA (2015). Phylesystem: a git-based data store for community-curated phylogenetic estimates. Bioinformatics.

[18] Smith SA, Beaulieu JM, Donoghue MJ (2009). Mega-phylogeny approach for comparative biology: an alternative to supertree and supermatrix approaches. BMC Evol Biol.

[19] Antonelli A, Hettling H, Condamine FL, Vos K, Nilsson RH, Sanderson MJ, Sauquet H, Scharn R, Silvestro D, Töpel M (2017). Toward a self-updating platform for estimating rates of speciation and migration, ages, and relationships of taxa. Syst Biol.

[20] Izquierdo-Carrasco F, Cazes J, Smith SA, Stamatakis A (2014). Pumper: phylogenies updated perpetually. Bioinformatics.

[21] Pearse WD, Purvis A (2013). phylogenerator: an automated phylogeny generation tool for ecologists. Methods Ecol Evol.

[22] Jones MR, Good JM (2016). Targeted capture in evolutionary and ecological genomics. Mol Ecol.

[23] Andermann T, Torres Jiménez MF, Matos-Martínez P, Batista R, Blanco-Pastor JL, Gustafsson ALS, Kistler L, Liberal IM, Oxelman B, Bacon CD, Antonelli A (2020). A guide to carrying out a phylogenomic target sequence capture project. Front Genetics.

[24] Fragoso-Martínez I, Salazar GA, Martínez-Gordillo M, Magallón S, Sánchez-Reyes L, Lemmon EM, Lemmon AR, Sazatornil F, Mendoza CG (2017). A pilot study applying the plant Anchored Hybrid Enrichment method to New World sages (Salvia subgenus Calosphace, Lamiaceae). Mol Phylogenetics Evol.

[25] Piel W, Chan L, Dominus M, Ruan J, Vos R, Tannen V. Treebase v. 2: a database of phylogenetic knowledge. e-Biosphere. London. 2009.

[26] Vos RA, Balhoff JP, Caravas JA, Holder MT, Lapp H, Maddison WP, Midford PE, Priyam A, Sukumaran J, Xia X (2012). NeXML: rich, extensible, and verifiable representation of comparative data and metadata. Syst Biol.

[27] Piel WH, Vos RA. Treebasedmp: a toolkit for phyloinformatic research. bioRxiv, 399030. 2018.

[28] Morrison DA (2006). Multiple sequence alignment for phylogenetic purposes. Aust Syst Bot.

[29] Thénault, Sylvain (Logilab S.A.): Pylint. Accessed March 2021. https://www.pylint.org/.

[30] Thénault, Sylvain (Logilab S.A.), PyCQA, and contributors: Pylint User Manual. Accessed March 2021. http://pylint.pycqa.org/en/latest/.

[31] OpenTreeOfLife, Redelings B, Cranston KA, Allman J, Holder MT, McTavish EJ. Open tree of life taxonomy V. 3.2. https://tree.opentreeoflife.org/about/taxonomy-version/ott3.2.

[32] OpenTreeOfLife: Name Resolution (TNRS) bulk mapping tool. https://tree.opentreeoflife.org/curator/tnrs/.

[33] OpenTreeOfLife, McTavish EJ, Hinchliff CE, Allman JF, Brown JW, Cranston KA, Holder MT, Rees JA, Smith SA. Phylesystem’s top-level repository in the Open Tree of Life phylogenetic study document store. https://github.com/opentreeoflife/phylesystem

[34] Piel W, Chan L, Dominus M, Ruan,J. Vos R, Tannen V. TreeBASE: a database of phylogenetic knowledge. https://treebase.org/treebase-web/home.html.

[35] Vos, R.: SuperTreeBASE: data dump and code to summarize TreeBASE. https://github.com/TreeBASE/supertreebase.

[36] Altschul SF, Gish W, Miller W, Myers EW, Lipman DJ (1990). Basic local alignment search tool. J Mol Biol.

[37] OpenTreeOfLife, Rees JA, Cranston K. OpenTree’s taxonomic MRCA API. https://github.com/OpenTreeOfLife/germinator/wiki/Taxonomy-API-v3#mrca.

[38] Camacho C, George C, Vahram A, Ning M, Jason P, Kevin B, Thomas L (2009). BLAST+: architecture and applications. BMC Bioinform.

[39] Cock PJ, Antao T, Chang JT, Chapman BA, Cox CJ, Dalke A, Friedberg I, Hamelryck T, Kauff F, Wilczynski B (2009). Biopython: freely available Python tools for computational molecular biology and bioinformatics. Bioinformatics.

[40] The BioPython Contributors (1999–2018): BioPython 1.71, Module Bio.Blast.NCBIWWW. Accessed April 19, 2018. https://biopython.org/DIST/docs/api/Bio.Blast.NCBIWWW-module.html.

[41] Edgar RC (2004). Muscle: multiple sequence alignment with high accuracy and high throughput. Nucl Acids Res.

[42] Stamatakis A (2014). Raxml version 8: a tool for phylogenetic analysis and post-analysis of large phylogenies. Bioinformatics.

[43] Sukumaran J, Holder MT (2010). DendroPy: a Python library for phylogenetic computing. Bioinformatics.

[44] Redelings BD, Holder MT (2017). A supertree pipeline for summarizing phylogenetic and taxonomic information for millions of species. PeerJ.

[45] Gottlieb AM, Giberti GC, Poggio L (2005). Molecular analyses of the genus ilex (aquifoliaceae) in southern south america, evidence from aflp and its sequence data. Am Jo Bot.

[46] The Plant List 2013. Version 1.1: list of name records for the generic epithet *Ilex*. http://www.theplantlist.org/tpl1.1/search?q=ilex.

[47] Chase MW, Christenhusz M, Fay M, Byng J, Judd WS, Soltis D, Mabberley D, Sennikov A, Soltis PS, Stevens PF (2016). An update of the Angiosperm Phylogeny Group classification for the orders and families of flowering plants: APG IV. Bot J Linn Soc.

[48] Cuénoud P, Martinez M.A.d.P, Loizeay P.-A, Spichiger R, Andrews S, Manen J.-F (2000). Molecular phylogeny and biogeography of the genus *Ilex* L.(Aquifoliaceae). Ann Bot.

[49] Manen J-F, Barriera G, Loizeau P-A, Naciri Y (2010). The history of extant Ilex species (Aquifoliaceae): evidence of hybridization within a Miocene radiation. Mol Phylogenetics Evol.

[50] Setoguchi H, Watanabe I (2000). Intersectional gene flow between insular endemics of Ilex (Aquifoliaceae) on the Bonin Islands and the Ryukyu Islands. Am J Bot.

[51] Selbach-Schnadelbach A, Cavalli SS, Manen J-F, Coelho GC, De Souza-Chies TT (2009). New information for Ilex phylogenetics based on the plastid psbA-trnH intergenic spacer (Aquifoliaceae). Bot J Linn Soc.

[52] Yao X, Song Y, Yang J-B, Tan Y-H, Corlett RT (2020). Phylogeny and biogeography of the hollies (*Ilex* L., Aquifoliaceae). J Syst Evol.

[53] Gottlieb AM, Giberti GC, Poggio L. TreeBASE study 1091. https://treebase.org/treebase-web/search/study/summary.html?id=1091.

[54] Gottlieb AM, Giberti GC, Poggio L. Phylesystem study pg\_2827. https://tree.opentreeoflife.org/curator/study/edit/pg_2827/?tab=home.

[55] OpenTreeOfLife, Redelings B, Reyes LLS, Cranston KA, Allman J, Holder MT, McTavish EJ. Open Tree of Life Synthetic subtree, node id mrcaott68451ott89474. https://tree.opentreeoflife.org/opentree/opentree12.3@mrcaott68451ott89474/Ilex-theizans--Ilex-dumosa.

[56] Yao X, Song Y, Yang J-B, Tan Y-H, Corlett RT. Phylesystem study ot\_1984. https://tree.opentreeoflife.org/curator/study/view/ot_1984.

[57] Yao X, Song Y, Yang J-B, Tan Y-H, Corlett RT. Phylogeny and biogeography of the hollies (*Ilex* L., Aquifoliaceae), Dryad, Dataset. https://datadryad.org/stash/dataset/10.5061/dryad.k0p2ngf4x.Accessed: April 2020.

[58] Berger SA, Krompass D, Stamatakis A (2011). Performance, accuracy, and web server for evolutionary placement of short sequence reads under maximum likelihood. Syst Biol.

[59] Matsen F, Kodner R, Armbrust EV (2010). pplacer: linear time maximum-likelihood and Bayesian phylogenetic placement of sequences onto a fixed reference tree. BMC Bioinform.

[60] Smith SA, Walker JF (2019). Pyphlawd: a python tool for phylogenetic dataset construction. Methods Ecol Evol.

[61] Bennett DJ, Hettling H, Silvestro D, Zizka A, Bacon CD, Faurby S, Vos RA, Antonelli A (2018). phylotar: an automated pipeline for retrieving orthologous dna sequences from genbank in r. Life.

[62] Huang H, Knowles LL (2009). What is the danger of the anomaly zone for empirical phylogenetics?. Syst Biol.

[63] Song S, Liu L, Edwards SV, Wu S (2012). Resolving conflict in eutherian mammal phylogeny using phylogenomics and the multispecies coalescent model. Proc Natl Acad Sci.

[64] Morel B, Barbera P, Czech L, Bettisworth B, Höbner L, Lutteropp S, Serdari D, Kostaki E-G, Mamais I, Kozlov AM, Pavlidis P, Paraskevis D, Stamatakis A (2020). Phylogenetic analysis of SARS-CoV-2 data is difficult. Mol Biol Evol.

[65] Zhu T, Yang Z (2021). Complexity of the simplest species tree problem. Mol Biol Evol.

[66] Mirarab S, Reaz R, Bayzid MS, Zimmermann T, Swenson MS, Warnow T (2014). ASTRAL: genome-scale coalescent-based species tree estimation. Bioinformatics.

[67] Chifman J, Kubatko L (2014). Quartet inference from SNP data under the coalescent model. Bioinformatics.

[68] Webb CO, Slik JF, Triono T (2010). Biodiversity inventory and informatics in Southeast Asia. Biodiver Conserv.

[69] San Mauro D, Agorreta A (2010). Molecular systematics: a synthesis of the common methods and the state of knowledge. Cell Mol Biol Lett.

[70] Helmus MR, Ives AR (2012). Phylogenetic diversity-area curves. Ecology.

[71] Stoltzfus A, Lapp H, Matasci N, Deus H, Sidlauskas B, Zmasek CM, Vaidya G, Pontelli E, Cranston K, Vos R (2013). Phylotastic! making tree-of-life knowledge accessible, reusable and convenient. BMC Bioinform.

[72] OpenTreeOfLife, Redelings B, Reyes LLS, Cranston KA, Allman J, Holder MT, McTavish EJ. Open tree of life synthetic subtree of the genus *Ilex*, Node Id Ott727571. https://tree.opentreeoflife.org/opentree/opentree12.3@ott727571/Ilex.

